# 泊马度胺治疗复发/难治性多发性骨髓瘤的疗效、安全性研究

**DOI:** 10.3760/cma.j.issn.0253-2727.2023.01.015

**Published:** 2023-01

**Authors:** 静 卢, 海燕 何, 璐 李, 琬婷 强, 进 刘, 沛 郭, 华 姜, 卫军 傅, 鹃 杜

**Affiliations:** 海军军医大学第二附属医院（上海长征医院）血液病科，全军骨髓瘤与淋巴瘤疾病中心，上海 200003 Department of Hematology, The Myeloma & Lymphoma Center, The Second Affiliated Hospital of Naval Medical University (Shanghai Changzheng Hospital), Shanghai 200003, China

多发性骨髓瘤（MM）是好发于老年患者的恶性浆细胞疾病。近10余年靶向新药（包括硼替佐米、来那度胺等）、造血干细胞移植及嵌合抗原受体T（CAR-T）细胞免疫治疗的应用，使骨髓瘤患者的疗效和预后取得了革命性进步[1]，但MM至今仍无法治愈，患者终将面临疾病复发进展，进而难治[2]。如何为复发/难治性MM（RRMM）患者选择治疗方案仍然是临床面临的难题。泊马度胺是第三代免疫调节剂，具有多重抗骨髓瘤机制，通过与Cereblon结合激活E3泛素连接酶复合物活性，促进底物蛋白Ikaros和Aiolos泛素化和蛋白水解，抑制MM细胞增殖[3]–[5]，并发挥免疫调节和抗血管生成作用，对既往硼替佐米及来那度胺等靶向药物耐药的MM患者有显著疗效。2013年泊马度胺被美国食品药品监督管理局（FDA）批准用于治疗RRMM，2020年11月泊马度胺在我国获批上市。目前泊马度胺在国内应用时间较短，其治疗RRMM的疗效、安全性数据较少。因此本研究回顾性分析50例应用泊马度胺治疗的RRMM患者的疗效及安全性，旨在为RRMM患者的治疗选择提供借鉴和参考。

## 病例与方法

1. 病例：回顾性分析2020年11月至2021年10月在上海长征医院血液科应用泊马度胺治疗的50例RRMM患者的疗效及安全性。患者既往接受至少一线治疗后疾病进展或复发难治，接受以泊马度胺为主的三药方案治疗至少2个疗程。每疗程评估泊马度胺的疗效、安全性及患者的预后。收集RRMM患者泊马度胺治疗前的基线指标，包括性别、年龄、M蛋白类型、DS（Durie-Salmon）分期、国际分期系统（ISS）分期、修订后的国际分期系统（R-ISS）分期、血红蛋白、β_2_-微球蛋白、血清乳酸脱氢酶、血肌酐、血清游离轻链（sFLC）和骨髓浆细胞水平等。通过间期荧光原位杂交（FISH）检测细胞遗传学特征，包括：IgH重排（阈值5％）、del（13q）（阈值20％）、1q21扩增（阈值20％）、del（17p）（阈值20％）、t（4;14）（阈值10％）、t（14;16）（阈值10％）、t（11;14）（阈值10％）。高危细胞遗传学异常（CA）的定义是FISH检测出del（17p）、t（4;14）、t（14;16）。

2. 治疗方案：50例RRMM患者均接受至少2个疗程以泊马度胺为基础的三药联合治疗，具体方案如下：①以泊马度胺联合烷化剂为基础的方案：PCd（泊马度胺+地塞米松+环磷酰胺）方案20例（40.0％），具体为泊马度胺4 mg/d，第1～21天；地塞米松20 mg，每周2次；环磷酰胺200 mg/m^2^，每周1次。②泊马度胺联合蛋白酶体抑制剂：PVd（泊马度胺+硼替佐米+地塞米松）方案20例（40.0％），具体为：泊马度胺4 mg/d，第1～21天；硼替佐米1.3 mg·m^−2^·d^−1^，第1、4、7、10天；地塞米松20 mg/d，第1、2、4、5、7、8、10、11天。③泊马度胺联合CD38单抗为基础的方案：DPd（达雷妥尤单抗+泊马度胺+地塞米松）方案10例（20.0％），具体为：达雷妥尤单抗16 mg/kg，第1、2疗程的第1、8、15、22天，第3、4疗程的第1、15天，第5疗程及以后每月1次；泊马度胺4 mg/d，第1～21天；地塞米松20 mg/d，第1、2、4、5、7、8、10、11天。

患者出现3级以上血液学不良反应时，暂停化疗并予对症支持治疗，不良反应消失后，泊马度胺剂量调整至2 mg/d，继续治疗1个疗程。如后续未发生3级以上不良反应，可尝试恢复泊马度胺剂量至4 mg/d。如下个疗程患者仍出现3级以上不良反应，可将三药联合方案中除泊马度胺和地塞米松以外的化疗药物（如环磷酰胺、硼替佐米、达雷妥尤单抗）在每疗程按总剂量的25％、50％依次进行剂量下调，如仍有3级以上不良反应，考虑调整为其他化疗方案。在患者入院后常规进行血栓筛查，按照《2020美国血液病学会深静脉血栓及肺动脉栓塞指南》[6]进行评估，根据患者病情及个人情况，选择口服阿司匹林100 mg/d或利伐沙班10 mg/d预防深静脉血栓形成，患者出现临床症状后完善血管彩超。

3. 疗效评估及不良反应：MM的诊断及疗效评估标准参照2020年MM诊治指南[7]，疗效分为严格意义的完全缓解（sCR）、完全缓解（CR）、非常好的部分缓解（VGPR）、部分缓解（PR）、微小缓解（MR）、血液学稳定（SD）及疾病进展（PD）。总缓解率（ORR）为sCR率、CR率、VGPR率及PR率之和。治疗相关不良反应评估按照美国常见不良反应术语评定标准5.0版进行评定。

4. 随访：随访方式为查阅患者的住院记录、门诊病历及电话随访，随访截止时间为2022年3月22日。中位随访时间9.0（1.9～16.8）个月。疾病无进展生存（PFS）时间定义为开始泊马度胺治疗至疾病进展或末次随访时间，总生存（OS）时间定义为患者开始泊马度胺治疗至因任何原因死亡的时间。

5. 统计学处理：采用SPSS 25.0软件进行统计学分析，R语言4.1.3软件作图，连续变量以中位数（范围）形式描述，分类变量以例数（百分比）形式描述，采用Kaplan-Meier法分析OS和PFS，*P*<0.05为差异有统计学意义。

## 结果

1. 临床特征：其中年龄>65岁患者23例（46％），中位年龄为63（44～79）岁，男30例（60％）。32例（64％）患者HGB<100 g/L，5例（10％）患者血肌酐≥2 g/L，31例（62％）患者β_2_-微球蛋白≥3.5 mg/L，26例（52％）患者乳酸脱氢酶≥245 U/L。所有患者均为DS分期Ⅲ期，其中38例（76％）患者为ⅢA期，12例（24％）患者为ⅢB期；ISS分期Ⅰ期、Ⅱ期、Ⅲ期患者分别为3例（6％）、19例（38％）和28例（56％）；R-ISS分期Ⅰ期、Ⅱ期、Ⅲ期患者分别为1例（2％）、32例（64％）和17例（34％）。M蛋白类型中IgG型23例（46％），IgA型10例（20％），IgD型9例（18％），轻链型8例（16％）。11例（22％）患者伴高危CA。21例（42％）患者合并一处或多处髓外病变，其中15例伴骨旁侵犯（累及胸椎、髂骨、肋骨等），3例伴中枢侵犯，2例伴肝脏侵犯，3例伴胸膜侵犯，1例伴左下肢软组织侵犯，1例伴齿龈侵犯。FISH检测示：del（17p）7例（14％），t（4;14）6例（12％），1q21扩增36例（72％）。前期中位治疗线数为3（1～7）线，从诊断至开始接受泊马度胺治疗的中位时间为63.4（2.5～174.9）个月。16例（32％）患者既往进行了自体造血干细胞移植。50例患者中40例（80％）既往对来那度胺耐药，36例（72％）既往对硼替佐米耐药，15例（30％）既往对CD38单抗耐药，4例（8％）既往对CAR-T细胞免疫治疗耐药。单药耐药患者13例，两药耐药患者16例，三种及以上药物耐药患者21例。前线治疗最佳疗效为sCR、CR、VGPR、PR、<PR者分别为9、10、15、13、3例。

2. 疗效评估：50例RRMM患者接受至少2个疗程以泊马度胺为基础的三药方案治疗，中位接受4（2～13）个疗程治疗，中位随访时间9（1.9～16.8）个月。ORR为68％，具体为：2例PD，9例SD，5例MR，23例PR，6例VGPR，3例CR，2例sCR。

亚组分析显示，伴高危CA［伴17p−、t（4;14）］患者11例，PCd方案6例、PVd方案3例、DPd方案2例，ORR为36.3％；不伴高危CA患者39例，PCd方案17例、PVd方案14例、DPd方案8例，ORR达76.9％，组间比较差异有统计学意义（*P*＝0.016）。伴髓外病变患者21例，PCd方案9例、PVd方案6例、DPd方案6例，ORR为71.4％；无髓外病变患者29例，PCd方案11例、PVd方案14例、DPd方案4例，ORR为65.5％，组间差异无统计学意义（*P*＝0.449）。PCd组患者20例，ORR为60.0％；PVd组患者20例，ORR为70.0％；DPd组患者10例，ORR为80.0％，三组间ORR的差异无统计学意义（*P*＝0.537）。不同耐药患者疗效分析：来那度胺耐药组患者40例，PCd方案17例、PVd方案14例、DPd方案9例，ORR为65.0％；硼替佐米耐药组患者36例，PCd方案15例、PVd方案13例、DPd方案8例，ORR为77.0％；CD38耐药组患者15例，PCd方案5例、PVd方案7例、DPd方案3例，ORR为60.0％；CAR-T细胞耐药患者4例，PCd方案2例、PVd方案1例、DPd方案1例，其中2例获得PR，2例获得CR。

3. 生存分析：50例RRMM患者的中位PFS时间为9.3个月，1年PFS率为39.2％，中位OS时间为15.0个月，1年OS率为65.5％。

伴高危CA组与不伴高危CA组患者的中位PFS时间分别为8.8个月和9.4个月，1年PFS率分别为38.2％和39.0％（*P*＝0.804），中位OS时间分别为未达到和14.9个月，1年OS率分别为70.1％和62.1％（*P*＝0.905）。伴髓外病变组与无髓外病变组的中位PFS时间分别为6.6个月和9.4个月，1年PFS率分别为32.7％和42.8％（*P*＝0.325），中位OS时间分别为14.9个月和未达到，1年OS率分别为61.0％和61.5％（*P*＝0.331）。PCd组、PVd组和DPd组患者中位PFS时间分别为9.0个月、8.8个月和14.3个月，1年PFS率分别为26.4％、38.9％和58.3％（*P*＝0.471），1年OS率分别为57.1％、63.3％和90.0％，中位OS时间均为未达到（*P*＝0.296），三组患者PFS和OS的差异均无统计学意义。

4. 安全性评估：本研究中，患者3级或4级不良反应发生率为38％。3级或4级白细胞减少5例（10％），3级或4级血小板减少4例（8％），3级或4级贫血6例（12％）。12例（24％）患者出现感染，其中6例（12％）出现肺部感染。1例患者出现3级腹泻。在积极预防深静脉血栓情况下，有1例（2％）患者发生下肢深静脉血栓。此外，2例患者出现皮疹伴瘙痒，抗过敏治疗后好转，1例患者出现失眠。未报告不良反应相关死亡。

## 讨论

泊马度胺是第三代免疫调节剂，对骨髓瘤细胞的抗增殖效应是沙利度胺的100倍、来那度胺的10倍[5]。2013年泊马度胺获得FDA批准用于治疗RRMM患者。泊马度胺联合低剂量地塞米松（Pd）方案治疗RRMM患者的疗效优于泊马度胺单药治疗，显著延长患者的PFS时间和OS时间。后续临床研究关注Pd方案联合其他药物治疗RRMM，如联合环磷酰胺、蛋白酶体抑制剂（包括硼替佐米、卡非佐米）、CD38单抗等。2020年11月泊马度胺在中国获批上市。为观察泊马度胺的疗效及预后，我中心对50例接受以泊马度胺为基础方案治疗的RRMM患者的疗效及预后进行回顾性分析。研究结果显示，50例RRMM患者的ORR达68％，中位PFS时间为9.3个月，中位OS时间为15.0个月，总体疗效与既往报道相近。泊马度胺治疗RRMM患者的安全性良好，最常见的3～4级不良反应为骨髓抑制和感染。本研究显示了以泊马度胺为基础的三药联合方案的有效性及安全性。

环磷酰胺是常用于MM治疗的经典烷化剂类药物。本研究中20例患者接受PCd方案治疗，该组ORR达60.0％，与既往报道类似。Baz等[8]在2016年率先报道了PCd方案的Ⅱ期临床试验结果，在至少接受二线治疗且对来那度胺耐药的RRMM患者中，ORR达64.7％，PFS时间为9.5个月。亚洲骨髓瘤工作网进行了一项前瞻性Ⅱ期研究（AMN001试验），纳入136例既往接受硼替佐米治疗且对来那度胺耐药或难治的RRMM患者，39例接受PCd方案治疗，ORR为43.6％，中位缓解持续时间为12.6个月，中位OS时间为16.3个月[9]。上述研究中PCd组患者ORR较本研究低，可能与药物可及性相关，上述研究中半数以上患者接受至少三线治疗，18.4％患者前期对卡非佐米、伊沙佐米、帕比司他、CD38单抗或依洛珠单抗耐药，接受自体造血干细胞移植比例（50％）也显著高于本研究（32％）。来自法国的真实世界研究显示，PCd方案治疗RRMM患者的ORR达76％，其中VGPR及以上的比例为27％，中位PFS时间为7.3个月，与本研究的疗效及预后相近[10]。本研究PVd治疗组中，20例患者ORR为70.0％，与Ⅲ期OPTIMISMM试验结果相近[11]。OPTIMISMM试验中PVd方案的ORR较Vd方案显著升高（82.2％对50.0％）。OPTIMISMM试验中亚洲患者的分析显示，中位随访14.8个月，12例接受PVd方案治疗的日本RRMM患者的ORR达100％，中位PFS时间为17.6个月[12]。2017年FDA批准CD38单抗与泊马度胺、地塞米松（DPd）联合治疗接受过来那度胺和蛋白酶体抑制剂治疗的MM患者。本研究10例接受DPd方案治疗患者的ORR达80.0％，略高于APOLLO研究中的69％[13]，可能与亚组样本量小、前期治疗不同、首次复发患者比例较高、纳入高龄患者少、患者地域差异等因素相关。

亚组分析显示，11例合并高危CA患者接受泊马度胺治疗的ORR仅为36.3％，明显低于不伴高危CA患者（76.9％）。尽管伴高危CA组患者数少，本研究仍提示DPd方案治疗可使高危患者获益。本研究中伴高危CA组与不伴高危CA组患者PFS与OS的差异无统计学意义，可能是由于部分治疗周期较短的患者尚未获得最佳缓解，还需要长期随访，延长观察时间。由于本研究为真实世界的回顾性研究，目前患者例数有限，且后续治疗及基线差异等均可影响预后，未来还需要进一步扩大样本量及前瞻性研究加以验证。伴髓外病变的MM患者预后不良，如何治疗是临床面临的难题。本研究中伴髓外病变的RRMM患者ORR达71.4％，提示泊马度胺联合化疗方案在伴髓外病变的MM患者中疗效较好。既往文献也有类似报道，6例伴髓外病变MM患者基于泊马度胺治疗的ORR达83％[14]。

在安全性方面，本研究中观察到最常见的3级以上不良反应为白细胞减少、血小板减少、贫血和感染。在积极预防的情况下，1例患者出现深静脉血栓，总体安全性良好。2017年Ⅰ/Ⅱ期临床试验评估泊马度胺治疗RRMM的安全性，最常见的3级以上不良反应为中性粒细胞减少、白细胞减少，10％患者发生深静脉血栓[15]。本研究不良反应发生情况基本与上述研究类似，但深静脉血栓发生率更低，可能与目前MM诊治中常规进行预防性抗凝治疗、本研究样本量较小相关。在韩国老年患者中进行的PCd方案研究中，3级以上不良反应主要为血液学不良反应及感染，发生率均高于本研究，可能与患者高龄、免疫力低相关[16]。

综上所述，以泊马度胺为基础的三药联合方案治疗RRMM患者的疗效及安全性良好，与既往国外报道结果类似，未来有待进一步行大样本前瞻性研究及长期随访，观察其远期疗效及安全性。
